# Characterization of a New and Efficient Polyvalent Phage Infecting *E. coli* O157:H7, *Salmonella* spp., and *Shigella sonnei*

**DOI:** 10.3390/microorganisms9102105

**Published:** 2021-10-06

**Authors:** Su-Hyeon Kim, Damilare Emmanuel Adeyemi, Mi-Kyung Park

**Affiliations:** 1 School of Food Science and Biotechnology, Kyungpook National University, Daegu 41566, Korea; 1sh_hs1@naver.com (S.-H.K.); atidamilare1@gmail.com (D.E.A.); 2 Food and Bio-Industry Institute, Kyungpook National University, Daegu 41566, Korea

**Keywords:** polyvalent phage, effectiveness, *Escherichia coli* O157:H7, *Salmonella*, *Shigella sonnei*

## Abstract

Ongoing outbreaks of foodborne diseases remain a significant public health concern. Lytic phages provide promising attributes as biocontrol agents. This study characterized KFS-EC3, a polyvalent and lytic phage, which was isolated from slaughterhouse sewage and purified by cesium chloride density centrifugation. Host range and efficiency of plating analyses revealed that KFS-EC3 is polyvalent and can efficiently infect *E. coli* O157:H7, *Salmonella* spp., and *Shigella sonnei*. KFS-EC3 had a latent time of 20 min and burst size of ~71 phages/infected cell. KFS-EC3 was stable and infectious following storage at a pH range of 3 to 11 and a temperature range of −70 °C to 60 °C. KFS-EC3 could inhibit *E. coli* O157:H7 growth by 2 logs up to 52 h even at the lowest MOI of 0.001. Genomic analysis of KFS-EC3 revealed that it consisted of 167,440 bp and 273 ORFs identified as functional genes, without any genes associated with antibiotic resistance, virulence, allergenicity, and lysogenicity. This phage was finally classified into the *Tequatrovirus* genus of the *Myoviridae* family. In conclusion, KFS-EC3 could simultaneously infect *E. coli* O157:H7, *S*. *sonnei*, and *Salmonella* spp. with the lowest MOI values over long periods, suggesting its suitability for simultaneous pathogen control in foods.

## 1. Introduction

Globally, foodborne pathogens are estimated to cause approximately 600 million cases of foodborne illnesses and 420,000 deaths annually [1]. According to the Centers for Disease Control and Prevention (CDC), approximately 48 million people get sick, 128,000 are hospitalized, and 3000 die from foodborne illnesses annually in the United States [2]. To date, more than 250 foodborne pathogens have been reported, and the major ones include *Escherichia coli*, *Salmonella*, *Shigella*, and *Campylobacter* due to their frequent outbreaks [3]. In particular, *E*. *coli*, *Salmonella*, and *Shigella*, as opportunistic pathogens belonging to the *Enterobacteriaceae* family, have genetic similarities in terms of taxonomy and GC contents that makes differentiating them using only 16S rRNA sequencing difficult [4]. For instance, *E*. *coli* and *Shigella* spp. have a narrow diversity (<1%) [5] and the DNA homology between *E*. *coli* and *Salmonella* spp. is reported to be ~80% [6,7]. In addition, their common symptoms of diarrhea, fever, and abdominal cramps are similar, and they manifest at low infective doses, specifically at <20 cells for *Salmonella* [8] and *E*. *coli* O157:H7 [9,10,11] and 10 to 100 cells for *Shigella* [12]. Furthermore, these pathogens are mainly associated with raw or undercooked meat, dairy products, and fresh produce, indicating that they coexist possibly in most foods [13,14,15,16,17,18]. Therefore, the simultaneous control of these pathogens is more desirable and effective for ensuring food safety.

Phages are viruses that only infect target bacteria, despite having a smaller size compared to bacteria (approximately 50 times smaller) [19]. Phages have been increasingly investigated as specific, natural, eco-friendly, and promising biocontrol agents against foodborne pathogens [20]. In particular, their ubiquitous existence in nature (10^31^ phages) and excellent target-dependent specificity have provided extensive investigation of phage from various sources such as sewage, human and animal feces, and foods [21]. Moreover, the unique characteristics of the phage life cycle, either lytic or lysogenic (temperate), are usually investigated for phage application [22,23]. The lytic phage attaches to host bacteria using its tail fiber and then injects its DNA into the bacteria. The DNA of a lytic phage is replicated rapidly, and viral proteins are synthesized, followed by assembly using the replication machinery of the host bacteria. The phages are finally released by lysing, which kills the host bacterial cells. In contrast, a lysogenic (temperate) phage can exist as a prophage within the host in a stable coexistence, without causing its lysis after the integration of phage DNA into the host cell chromosome. However, the lysogenic phage can undergo a lytic cycle for bacterial lysis after induction by unfavorable conditions such as environmental stress [23]. Thus, the lytic phage is preferable as a biocontrol agent due to the specific lysis ability against the target pathogen, either in a single species or in multiple species [24]. Furthermore, the FDA approved ListShield^TM^ (Intralytix Inc., Baltimore, MD, USA) as a “food additive” for the control of *Listeria monocytogenes* in ready-to-eat products in 2006. Since then, several commercial phage products including EcoShield^TM^ (Intralytix Inc.), SalmoFresh^TM^ (Intralytix Inc.), and ShigaShield^TM^ (Intralytix Inc.) have been developed, however, nearly all of them focused individually on only one bacterial genus [20]. Thus, a new approach is required to identify a phage capable of controlling the major foodborne pathogens simultaneously with one dosage.

The main road map for developing a practical phage is to isolate it and then evaluate its effectiveness including its safety prior to food application [25]. The effectiveness of phage applications is determined by phage specificity (narrow vs. broad), phage fecundity (adsorption time, eclipse time, latent time, and burst size), phages/bacteria ratio, and stability under various environmental conditions such as pH and temperature [26]. In addition, the phage safety should be confirmed to have the absence of any genes encoding virulence, antibiotic resistance, allergen, and lysogenic property in the genome [27]. Thus, an effective phage should be capable of lysing the target bacteria (not just one target) with a minimum phage concentration in a certain time period, even if placed in various environment conditions. In this study, a lytic and polyvalent KFS-EC3, capable of infecting *E*. *coli*, *Salmonella* spp., and *S. sonnei,* was isolated and purified from slaughterhouse sewage. The efficiency of KFS-EC3 was further assessed by efficiency of plating (EOP) analysis and challenge assay, and its novelty and safety were analyzed by genetic annotation and genome comparative analysis to ensure its potential application as a biocontrol agent in foods. 

## 2. Materials and Methods

### 2.1. Bacterial Strains and Growth Conditions

*E. coli* O157:H7 ATCC 10536 was used as an indicator strain for phage isolation and was also used with 56 other strains for the specificity analysis (Table 1). The bacterial strains in Table 1 were selected as they are majorly associated with foodborne outbreaks. Each of the bacterial strains was cultivated in 25 mL of tryptic soy broth (TSB, Difco Laboratories Inc., Sparks, MD, USA) at 37 °C for 16 h with shaking at 110 rpm. After washing with sterilized phosphate buffered saline (PBS, pH 7.4, Life Technologies Co., Carlsbad, CA, USA) and centrifuging at 7000× *g* for 4 min at 4 °C three times, the bacterial pellet was resuspended with PBS. The concentration of the bacterial suspension was adjusted to 10^8^ CFU/mL based on standard curves constructed by measuring optical density at 640 nm.

### 2.2. Isolation of Lytic Phage

Phage isolation was performed using the previously described method [21]. An aliquot of 25 mL of a slaughterhouse sewage sample (Daegu, Korea) was mixed with 225 mL of TSB containing 1 mL of *E. coli* O157:H7 ATCC 10536 and incubated at 37 °C for 16 h with shaking at 160 rpm. After centrifugation at 4000× *g* for 10 min at 4 °C, the supernatant was filtered using a 0.20-µm cellulose acetate filter (Advantec MFS Inc., Dublin, CA, USA). Next, 10 µL of the filtrate was drop-spotted on the surface of a tryptic soy agar (TSA, Difco Laboratories Inc., Sparks, MD, USA) plate overlaid with 4 mL of TA soft agar (4 g/L agar, 8 g/L nutrient broth, 5 g/L NaCl, 0.2 g/L MgSO_4_, 0.05 g/L MnSO_4_, and 0.15 g/L CaCl_2_) containing 200 µL of an overnight culture of *E. coli* O157:H7 ATCC 10536 to confirm phage presence (drop spot assay). Once a clear zone was observed, a plaque assay was performed to isolate a single plaque from it. One hundred microliters of phage filtrate were mixed with 4 mL of TA soft agar containing 200 µL of *E. coli* O157:H7 ATCC 10536 (8 Log CFU/mL). The mixture was poured onto TSA plates and incubated at 37 °C for 16 h. Each clear plaque was picked using a customized tip (made using a heated sterile surgical blade for increasing the bore size) and eluted into a sodium chloride-magnesium sulfate (SM) buffer (50 mM/L Tris-HCl, 100 mM NaCl, and 10 mM MgSO_4_, pH 7.5) with gentle agitation at 22 °C for 1 h.

### 2.3. Propagation and Purification of KFS-EC3

For high-titer phage propagation, 1% (*v*/*v*) of *E. coli* O157:H7 ATCC 10536 suspension was mixed with 3 mL of TA broth (8 g/L nutrient broth, 5 g/L NaCl, 0.2 g/L MgSO_4_, 0.05 g/L MnSO_4_, and 0.15 g/L CaCl_2_) to incubate at 37 °C for 2 h. Afterward, 1 mL of the eluted single plaque was added, and the mixture was further incubated for 2 h at 37 °C. Following centrifugation at 6000× *g* for 20 min at 4 °C, the supernatant was filtered using a 0.20-µm cellulose acetate filter. This procedure was repeated with increasing amounts of TA broth. The filtrate of the final propagated phage was precipitated using 10% (*w/v*) polyethylene glycol (PEG) 6000 (Sigma-Aldrich Co., St. Louis, MO, USA) and 10 mL of 1 M NaCl. After incubation at 4 °C for 16 h and centrifugation at 7200× *g* for 20 min at 4 °C, the obtained pellet was suspended in SM buffer. A CsCl gradient ultracentrifugation was then performed at 39,000× *g* for 2 h at 4 °C [18]. A bluish band layer was collected and dialyzed for 4 h in 600 mL of dialysis buffer (5 M NaCl, 1 M MgCl_2_, and 1 M Tris-HCl, pH 8.0) at 4 °C. Finally, the concentration of the purified phage (hereafter referred to as KFS-EC3) was measured using the plaque assay expressed as a plaque-forming unit (PFU)/mL. The phage was stored in a sterile glass vial at 4 °C for further characterization studies.

### 2.4. Morphological Analysis of KFS-EC3

The morphological characteristics of the purified KFS-EC3 were examined using transmission electron microscopy (TEM; HT7700, Hitachi Ltd., Chiyoda, Japan). On a copper grid, 10 µL of KFS-EC3 was deposited and incubated for 1 min at 22 °C. The phage on the grid was stained with 2% uranyl acetate (Sigma-Aldrich) for observation at 100 kV at 50,000–200,000× magnification.

### 2.5. Analysis of the Specificity and Efficiency of Plating of KFS-EC3

The specificity of KFS-EC3 was investigated against 57 strains (Table 1) with a drop spot assay. After incubation at 37 °C for 16 h, the formation of a clear zone was observed and expressed as either positive (“+”) for the presence of a clear zone or negative (“−”) for the absence of a clear zone. Following the formation of clear zones, the efficiency of plating (EOP) was determined by performing a plaque assay, and the EOP value was calculated by dividing the number of plaques on each bacterial strain by the number of plaques on the indicator strain. 

### 2.6. One-Step Growth Curve Analysis of KFS-EC3

One percent (*v*/*v*) of *E. coli* O157:H7 ATCC 10536 suspension was sub-cultured into fresh TSB and incubated until it reached 0.5 of OD_640nm_. KFS-EC3 was added at an MOI of 0.001 and incubated for 6 min at 37 °C to allow adsorption of KFS-EC3 to the bacterial cell (Supplementary method S1). The mixture was centrifuged at 11,400× *g* for 10 min at 4 °C to remove the unabsorbed phages in the mixture. The infected bacterial pellet was resuspended into the same volume of TSB and incubated at 37 °C for 1 h. Two sets of samples were collected at every 5 min interval for chloroform treated set and untreated set. To determine the length of the eclipse period, one set (chloroform-treated set) was mixed with 1% chloroform (*v*/*v*) to release the intracellular progeny phages, while the other set was left untreated (without exposure to 1% chloroform) to determine the length of the latent period. To determine the eclipse period, latent period, and burst size of KFS-EC3, a plaque assay was immediately performed using the serial dilution of the two sets. 

### 2.7. pH and Temperature Stabilities of KFS-EC3

The stability of KFS-EC3 was investigated by exposing it to various pH and temperatures conditions. To test its pH stability, 1 mL of KFS-EC3 was mixed with 9 mL of TSB, which was adjusted to various pH (1, 2, 3, 4, 5, 6, 7, 8, 9, 10, 11, and 12) and incubated at 22 °C for 1 h. To assess temperature stability, a mixture of 1 mL of KFS-EC3 and 9 mL of TSB (pH 7.3) was incubated at −70, −20, 4, 22, 37, 50, 60, 70, 80, and 90 °C for 1 h. After incubation, the phage titers were determined using a plaque assay.

### 2.8. In Vitro Bacterial Challenge Assay

An overnight culture of *E*. *coli* O157:H7 ATCC 10536 was added into 100 mL of fresh TSB medium (2% inoculum, *v*/*v*) and incubated at 37 °C with gentle shaking until it reached 0.5 of OD_640nm_ (~8 log CFU/mL). Afterwards, KFS-EC3 was diluted using SM buffer and added to the bacterial suspension at a 1:1 volume ratio to attain an MOI of 10, 1.0, 0.1, 0.01, and 0.001. The suspension was then incubated at 37 °C for 64 h with gentle shaking, and the viable bacteria number was counted at every 4 h interval using TSA plates.

### 2.9. Genome Sequencing and Annotation of KFS-EC3

The genomic DNA of KFS-EC3 was extracted and purified using a Phage DNA Isolation Kit (Norgen Biotek Corp. Thorold, ON, Canada). Whole-genome sequencing of the purified DNA was performed (Macrogen Inc., Seoul, Korea) using the paired-end Miseq sequencing platform (Illumina Inc., San Diego, CA, USA). The raw reads were trimmed using Trimmomatic [28] to eliminate the low-quality reads and adapter sequences. The de novo assembly of the qualified sequences was performed by various k-mer using the SPAdes genome assembler (Illumina). The open reading frames (ORFs) of the assembled sequence were predicted and annotated using the Rapid Annotations using Subsystems Technology (RAST) server [29] and BLASTP. The potential tRNA genes in the genome sequence were predicted using ARAGORN [30] and RNAmmer (version 1.2) [31] in the rapid prokaryotic genome annotation pipeline (Prokka) [32]. The complete genome sequence of KFS-EC3 was deposited in the GenBank database, under the nucleotide sequence accession number MZ065353. The novelty of phage was assessed based on its similarity to other most closely related phages from the NCBI BLASTN database (https://blast.ncbi.nlm.nih.gov/Blast.cgi?PAGE_TYPE=BlastSearch, 5 May 2021). 

### 2.10. Bioinformatics Analysis of KFS-EC3

The virulent genes and allergenic factors of KFS-EC3 were, respectively, investigated using VirulenceFinder-2.0 [33] and the allergen database (http://www.allergenonline.com, 5 May 2021), which was provided by the Food Allergy Research. Furthermore, the annotated genome sequence was verified against the Comprehensive Antibiotic Resistance Database (CARD) [34] and ResFinder 2.1 for any acquired antimicrobial resistance genes [35]. The genes associated with lysogenic property were confirmed using the PHASTER’s database [36]. A genome map was generated using Geneious version 11.1.5 (Biomatters Ltd., Auckland, New Zealand). Phylogenetic analysis of KFS-EC3, based on complete genome, was conducted using the Virus Classification and Tree building Online Resource (VICTOR) [37]. In addition, two other phylogenetic trees of KFS-EC3 were constructed using the neighbor-joining method, with 1000 bootstrap values through MEGA X [38] based on the capsid protein and terminase large subunit of its most closely-related phages and type phages of other genera. Their complete genes and amino acid sequences were obtained from the NCBI database and analyzed via the ClustalW algorithm [39], a multiple alignment program, and Mega X. The average nucleotide identity (ANI) value was determined using the OrthoANI Tool v0. 93. 1 [40] based on the complete genome. Heat map analysis was conducted using the GraphPad Prism (GraphPad, San Diego, CA, USA). Finally, the genome of KFS-EC was compared with that of T4 phage and vB_EcoM_IME339 phage using Easyfig v2. 2. 3 [41].

### 2.11. Statistical Analysis

All the experiments were conducted in triplicates. The data were shown as the mean ± standard deviation. Statistical analysis of the data was performed using GraphPad Prism and InStat V.3 (GraphPad, San Diego, CA, USA). The means were compared using the student’s paired t-test for two-group comparisons and one-way analysis of variance (ANOVA) for multi-group comparisons. A *p*-value of less than 0.05, 0.01, or 0.001 indicated statistical significance.

## 3. Results

### 3.1. Isolation, Purification, and Morphological Analysis of KFS-EC3

**Figure 1:**
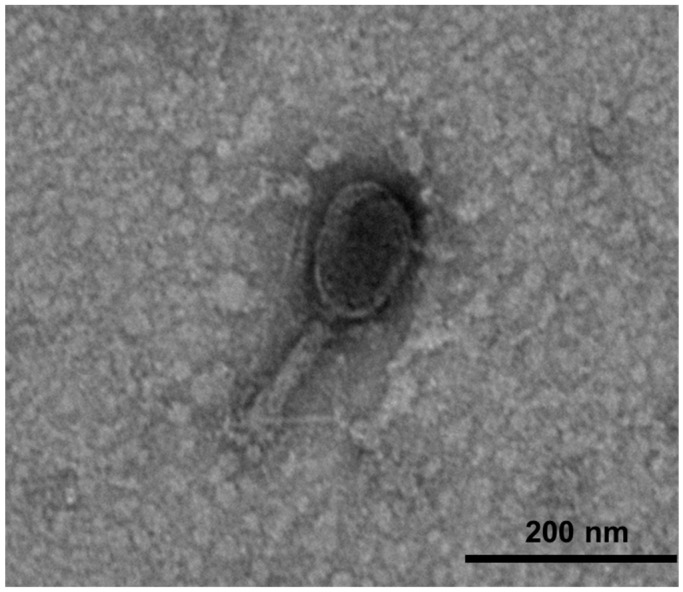
TEM images of KFS-EC3 at a magnification of ×25.0 k.

Only one lytic phage was isolated from a slaughterhouse sewage by single plaque isolation from the best clear plaque (Figure S1). This isolated phage was propagated and purified to a final concentration of (1.23 ± 0.18) × 10^10^ PFU/mL. The phage was named KFS-EC3, according to the nomenclature recommended by Ackermann, that the phage’s name should contain the first two letters of the genus and species of the indicator strain [42]. KFS-EC3 (Figure 1) was revealed to consist of an icosahedral head with a length of 96.35 ± 6.84 nm and width of 86.83 ± 7.52 nm, and a contractile tail of 68.42 to 133.48 nm in length, based on the analysis of 20 different TEM images of KFS-EC3. Thus, KFS-EC3 was shown to belong to the myovirus.

### 3.2. Specificity and EOP Analysis of KFS-EC3

The specificity of KFS-EC3 was investigated with 57 bacterial strains (Table 1). KFS-EC3 was able to infect seven bacterial strains, including all the three strains of *E. coli* O157:H7 (*E. coli* O157:H7, *E. coli* O157:H7 ATCC 10536, and *E. coli* O157:H7 204p), three strains of *Salmonella* spp. (*S.* Enteritidis, *S.* Mission, and *S.* Senftenberg), and *S. sonnei* ATCC 9290. However, it could not infect other strains of *E. coli*, *Salmonella*, *Shigella*, as well as other bacterial genera (*Aeromonas*, *Bacillus*, *Klebsiella*, *Listeria*, *Pseudomonas*, *Staphylococcus*, *Vibrio*, and *Yersinia*). EOP analysis was performed to relatively compare the lytic activity among seven susceptible bacterial strains, and all EOP values were greater than 0.5, indicating a high efficiency of infection [43]. The lytic activity of KFS-EC3 among the three bacterial species was ranked from high to low in order of *E. coli* O157:H7, *S. sonnei*, and *Salmonella* spp. Thus, KFS-EC3 exhibited a polyvalent lytic activity, implying its potential control of strains of bacteria in three genera including *E. coli* O157:H7, *S*. *sonnei*, *S.* Enteritidis, *S.* Mission, and *S.* Senftenberg.

### 3.3. Eclipse Time, Latent Period, and Burst Size of KFS-EC3

**Figure 2:**
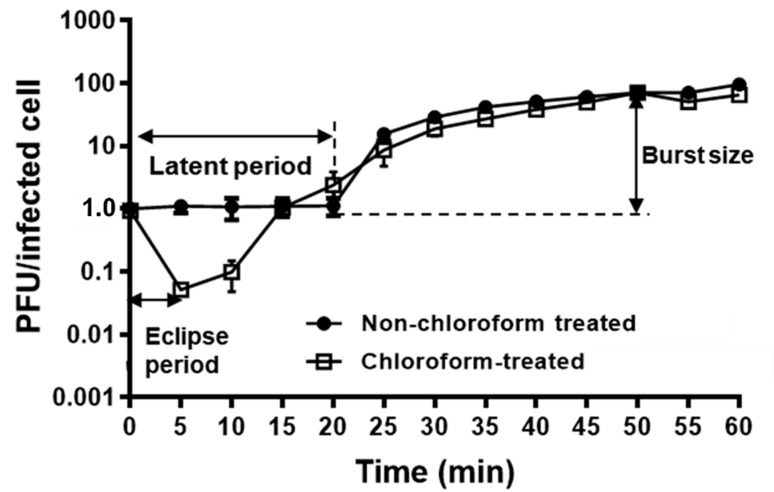
One-step growth curve analysis of KFS-EC3.The mixture of *E*. *coli* O157:H7 ATCC 10536 and KFS-EC3 at MOI of 0.001 was incubated for 1 h at 37 °C. A sample collected at every interval was treated with 1% chloroform (open square) while the other sample was not treated with chloroform (closed circle).

A one-step growth curve of KFS-EC3 was conducted to elucidate its lytic cycle and phage fecundity on the indicator strain, *E. coli* O157:H7 ATCC 10536 (Figure 2). According to the results from the adsorption assay (Figure S2), ~99% of the phage was adsorbed to the indicator strain after 6 min of infection. Afterwards, the phage eclipse time was observed to be 5 min. The latent period, the time taken by the phage to reproduce inside an infected bacterial cell, was determined to be 20 min, and the burst size was calculated to be (71 ± 0.05) PFU per infected cell.

### 3.4. Stability of KFS-EC3

**Figure 3:**
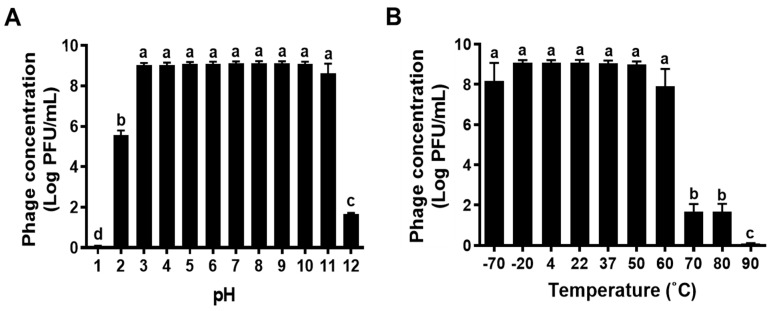
The stability of KFS-EC3 at various (**A**) pHs and (**B**) temperatures. Different letters (a–d) on the bars of either pH or temperature values indicate that the mean values differ significantly by one-way ANOVA test (*p* < 0.05).

When KFS-EC3 is used as a biocontrol agent in foods, it will be exposed to diverse environmental conditions. Thus, it would be important to study the lytic stability of KFS-EC3 at various pH levels and temperatures. KFS-EC3 exhibited excellent stability at the pH range of 3 to 11, however, its stability was significantly reduced to (5.56 ± 0.03) log PFU/mL and (1.65 ± 0.07) log PFU/mL at pH 2 and 12, respectively, from ~9.10 log PFU/mL (*p* < 0.05) (Figure 3A). In addition, it lost almost all infectivity at pH 1 with a reduction to (0.09 ± 0.01) log PFU/mL. Furthermore, KFS-EC3 exhibited robust stability at a broad temperature range between −70 °C and 60 °C (Figure 3B). Afterwards, its stability significantly decreased to (1.67 ± 0.39) log PFU/mL at both 70 °C and 80 °C, and its lytic activity was completely lost at 90 °C (*p* < 0.05).

### 3.5. In Vitro Bacterial Challenge Assay of KFS-EC3

**Figure 4:**
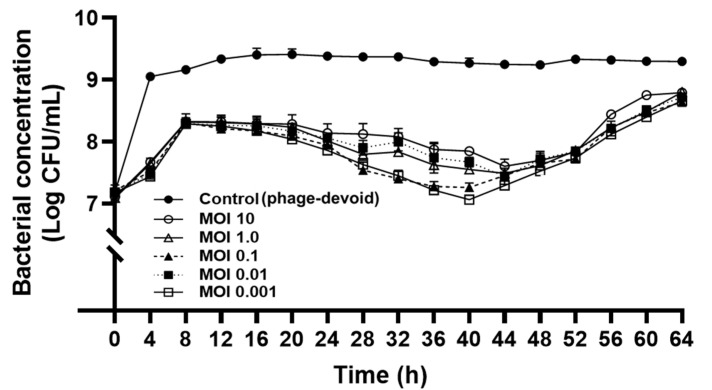
Bactericidal effect of KFS-EC3. An overnight culture of *E*. *coli* O157:H7 ATCC 10536 was infected with various concentrations of KFS-EC3 at a MOI of 10 (open circle), 1.0 (open triangle), 0.1 (closed triangle), 0.01 (closed square), and 0.001 (open square), respectively.

The lytic activity of KFS-EC3 at various MOIs was compared during a 64-h incubation by counting the viable bacterial cells (Figure 4). The period of incubation was stopped at 64 h because the phage treatment at the different MOIs attained the same growth level as that of the control after the 64 h timepoint. After 4 h of phage treatment, there was a significant reduction of the concentration of *E. coli* O157:H7 by a range of ~1.4 –1.6 logs, depending on various MOIs when compared to the control (phage-devoid). Interestingly, KFS-EC3 showed the greatest lytic activity at the lowest MOI of 0.001 (*p* < 0.05). Although there was slight bacterial regrowth for up to 8 h of incubation independent of MOI values, their growth was significantly lower than that of the control (*p* < 0.05). Interestingly, there was a significant reduction in the growth of *E. coli* O157:H7 up to 2 logs after 8 h of incubation, and it was sustained for 52 h at all MOIs (*p* < 0.05). More importantly, at the lowest MOI of 0.001, KFS-EC3 exhibited greater lytic activity than all other MOIs and sustained the activity throughout the incubation period. Thus, KFS-EC3 exhibited adequate efficiency and potential as an effective biocontrol agent since it could effectively control *E*. *coli* O157:H7 using only a low concentration of the phage for a long period.

### 3.6. Sequencing and Annotation of KFS-EC3 Genome

**Figure 5:**
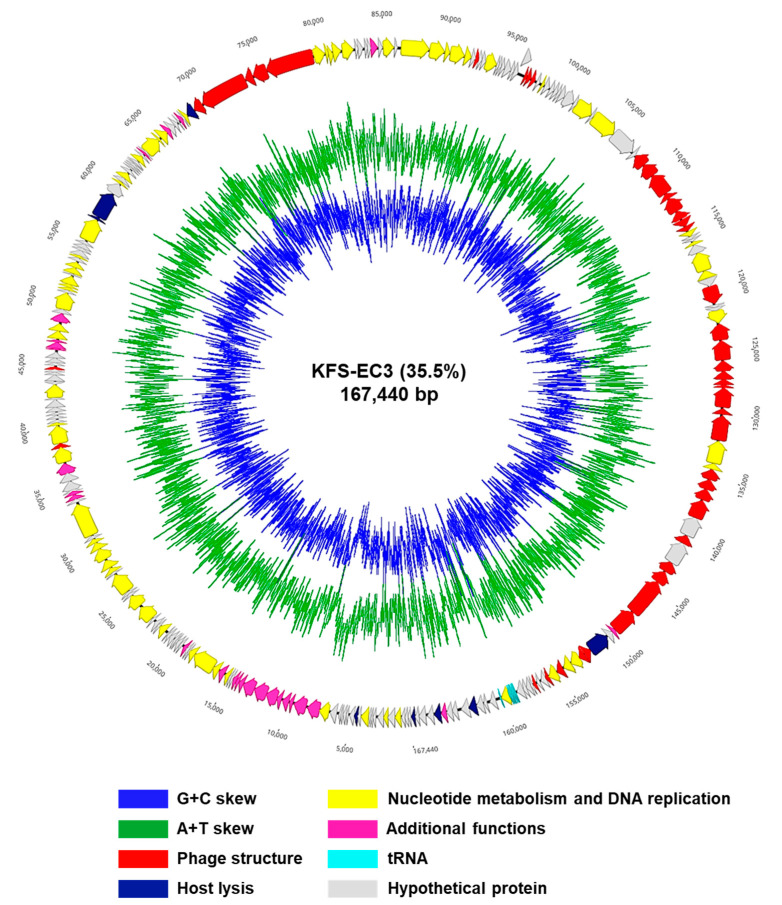
The genome map of KFS-EC3. The predicted ORFs (arrows) and direction of transcription (arrowheads) are indicated. The ORFs classified by the subsystem of the RAST pipeline were denoted as phage structure (red arrows), host lysis (navy), nucleotide metabolism and DNA replication (yellow), additional functions (pink), tRNA (sky blue), and hypothetical proteins (gray).

The sequencing of the KFS-EC3 genome produced 20,866,184 reads, which were trimmed to 18,852,944 reads. According to the de novo assembly, KFS-EC3 consisted of 167,440 bp with a 35.5% GC contents (Figure 5). In the analysis of annotation and visualization, a total of 273 ORFs and 8 tRNAs were predicted in the KFS-EC3 genome (Figure 5). Among these predicted 273 ORFs, 151 ORFs (55.68%) were annotated and organized into four functional groups, including constructing phage structure (the baseplate, neck, tail, tail fiber, and capsid protein), cell lysis (holin and lysozyme), nucleotide metabolism and DNA replication (terminase, RNA polymerase, DNA helicase, DNA polymerase, exonuclease, DNA ligase, and DNA helicase), and additional functions (glutaredoxin, thioredoxin, and phospholipase). In contrast, 122 ORFs (44.69%) were assumed to be hypothetical proteins with unknown functions due to the absence of identical genes in the NCBI database. More importantly, KFS-EC3 did not contain any genes associated with virulent factors from the database of VirulenceFinder-2.0 [34], antibiotic resistance from the databases of CARD [35] and ResFinder 2.1 [36], lysogenic activity from PHASTER’s database [37], or allergenicity from the allergen database (http://www.allergenonline.com, 5 May 2021). Overall, the genetic analysis suggests that KFS-EC3 would be a safe biocontrol agent for food application.

### 3.7. Phylogenetic and Genome Comparative Analysis of KFS-EC3

**Figure 6:**
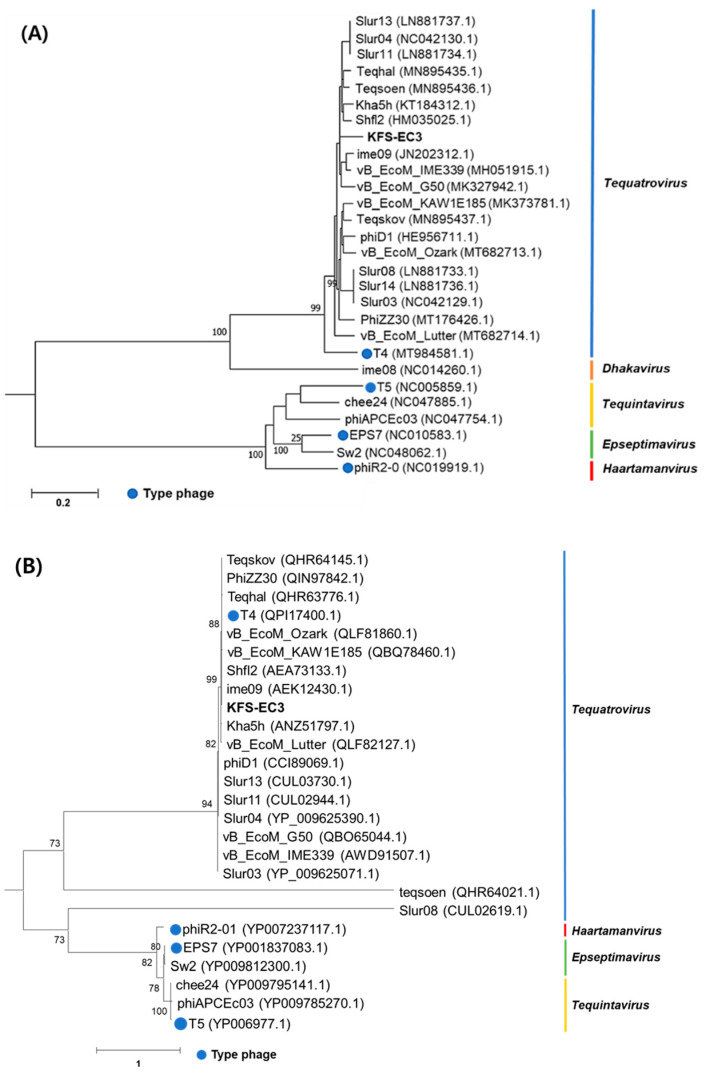

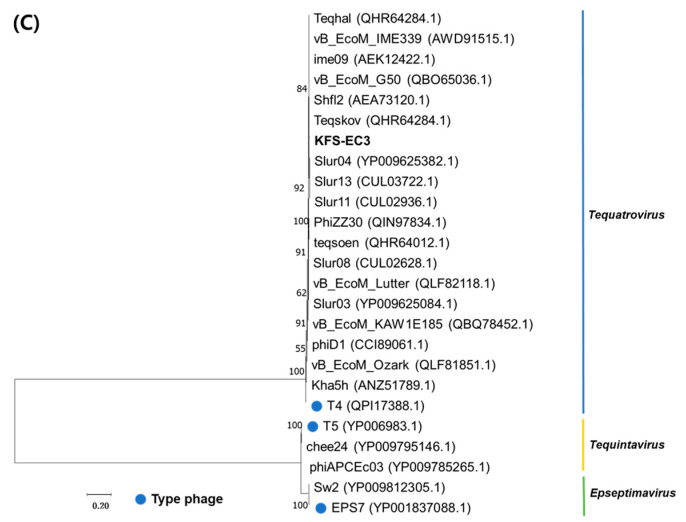
Phylogenic analysis of KFS-EC3 and other related phages based on the (**A**) complete genomes, (**B**) capsid protein, and (**C**) terminase large subunit.

**Figure 7:**
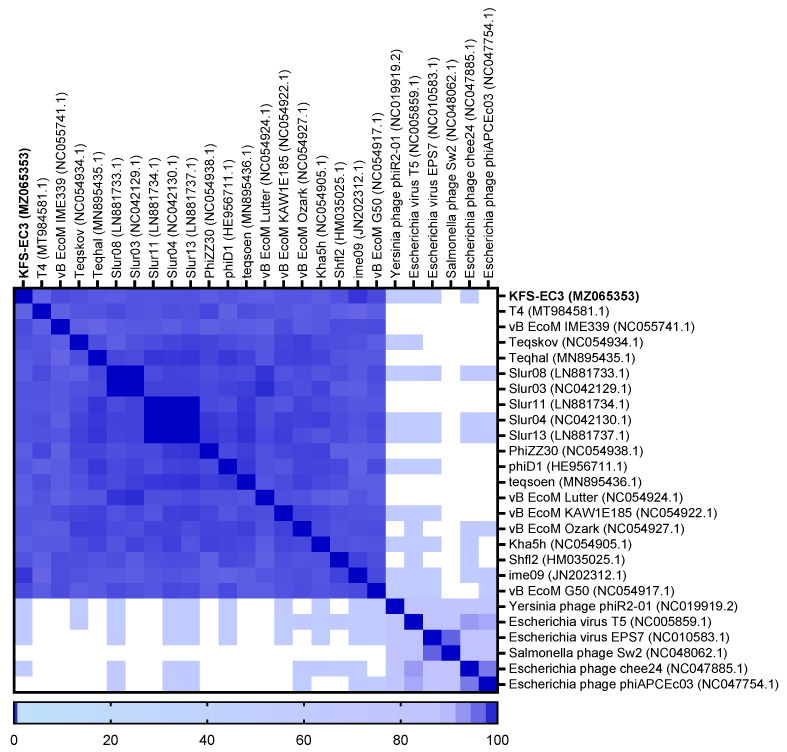
Heatmap analysis of the average nucleotide identity values of KFS-EC3 and other related phages in the NCBI database. Deeper shades of blue indicate a closer relationship.

**Figure 8:**
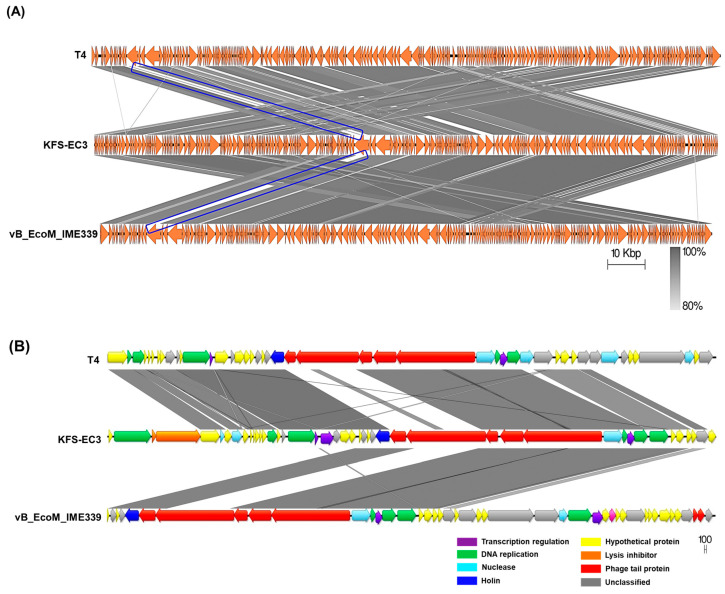
Genomic comparative analysis of KFS-EC3 (middle), *Escherichia* phage T4 (top), and *E*. *coli* phage vB_EcoM_IME339 (bottom). Gene clusters whose translated products shared low similarity were indicated using blue boxes. (**A**) Complete genome of the three phages visualized using Easyfig. (**B**) Boxed gene clusters at high magnification. Homologous regions were indicated by gray shading in figures.

The classification of KFS-EC3 was confirmed by phylogenetic tree analysis on the basis of the complete genome and major capsid protein [44]. From the complete genome, KFS-EC3 was clustered with ten phages of the *Tequatrovirus* genus including slur13, slur04, slur11, teqhal, teqsoen, Kha5h, Shfl2, ime09, vB_EcoM_IME339, and vB_EcoM_G50 (Figure 6A). With respect to the phylogenetic analysis based on capsid protein, KFS-EC3 was located in a single branch with eight *Tequatrovirus* phages (Figure 6B). Thus, considering the genetic relationships, this phage belongs to the *Tequatrovirus* genus of the *Myoviridae* family. Based on the large subunit of terminase, the phylogenetic analysis revealed that KFS-EC3 was clustered with teqhal, vB_EcoM _IME339, ime09, vB_EcoM_G50, Shfl2, and teqskov, suggesting a common DNA packaging process (Figure 6C). A total of 26 phages were selected based on ICTV and NCBI databases for heatmap analysis of the ANI value, including 19 *Tequatrovirus* phages, 3 *Tequintavirus* phages, 2 *Epseptimavirus* phages, and 1 *Haartmanvirus* phage. The heatmap and ANI analysis showed that KFS-EC had close relatedness with the other phages of a common cluster at the nucleotide level, and it was particularly similar to ime09 (97.13%), vB_EcoM_G50 (96.1%), and vB_EcoM_IME399 (96.04%), as shown by their ANI values that were above 96% (Figure 7). To further investigate the novelty of KFS-EC3, its genome was compared with that of vB_EcoM_IME399, which showed high similarity in all analyses, and with that of T4 phage, which is type phage of *Tequatrovirus* (Figure 8). The comparative analysis revealed that the genes of KFS-EC3 maintained a conserved gene orientation. The major difference between KFS-EC3 and the other phages was the low sequence similarity (<80%) of the tail protein (Figure 8B). Overall, these findings confirm that KFS-EC3 is distinctly genetically different from previously identified phages, and it was therefore classified into the *Tequatrovirus* genus of the *Myoviridae* family as a new phage.

## 4. Discussion

Foodborne illnesses caused by *E. coli* O157:H7, *Salmonella*, or *Shigella* are of great concern to public health. Lytic phages as biocontrol agents have garnered growing interest due to their bacterial lytic, physiologically robust, safe, and eco-friendly properties [20,45,46]. For developing a phage-based biocontrol agent, phage effectiveness should be ensured prior to its employment [47]. Most importantly, the specificity, high fecundity, and efficiency of the phage are crucial factors to determine the suitability of the isolated phage as a biocontrol agent [44,48].

Depending on the host range spectrum, phages are broadly categorized into either narrow (specific to a single bacterial genus) or polyvalent phages (broad, specific to more than two genera [49,50,51]). Despite the necessity of polyvalent phages for practical purposes, the majority of phages reported exhibit narrow specificity, targeting only one species (or just one strain) [20,45,52,53]. To the best of our knowledge, relatively few studies have reported results on polyvalent phages, and these include four myophages (SFP10 specific to *Salmonella* and *E*. *coli* [11], HY01 specific to *Shigella* and *E*. *coli* [9], PS5 specific to *Salmonella* and *E*. *coli* [44], and vB_EcoM_swi3 specific to *Salmonella* and *E*. *coli* [54]), as well as three siphophages (PEf1 specific to *Pseudomonas* and *E*. *coli* [55], phiC119 specific to *E*. *coli* and *Salmonella* [56], and SH6 specific to *E*. *coli* and *Shigella* [57]). All these polyvalent phages were specific to two genera, one of which was *E*. *coli*. Recently, three genus-level-specific polyvalent phages were reported, including the EcS1 myophage infecting *E*. *coli*, *S.* Enteritidis, and three strains of *Shigella (S. sonnei*, *S*. *boydii*, and *S*. *flexneri*) [58], and the SH7 myophage specific to *E*. *coli*, *S.* Paratyphi, and two strains of *Shigella* (*S*. *flexneri* and *S*. *dysenteriae*) [57], which confirmed that myophages often exhibit a broader host range than siphophages and podophages. Similar to the polyvalent EcS1 and SH7 phages, KFS-EC3 was found to infect three genera, namely *E. coli* O157:H7, three strains of *Salmonella* spp. (*S.* Enteritidis, *S.* Mission, and *S.* Senftenberg), and *S. sonnei*. Interestingly, KFS-EC3 exhibited high EOP values (>0.5) against all the host strains including *E. coli* O157:H7, *S*. *sonnei*, and three strains of *Salmonella,* whereas the polyvalent SFP10 showed low EOP values (<0.2) against *S*. Paratyphi among all the tested host stains (*S*. Typhimurium, *S*. Enteritidis, *S*. Dublin, and *E*. *coli*) [11]. For the relative comparison of bactericidal capacity of polyvalent KFS-EC3, the phage was exposed to each single strain of *E*. *coli* ATCC 10536, *S. sonnei* ATCC 9290, and *S.* Mission (used as a representative of *Salmonella* spp.), and their bacterial cocktail (Supplementary method S2). As shown in Figure S3, no significant differences were observed in bacterial reduction capacity among all the treatments, thus KFS-EC3 exhibited a similar polyvalent capacity when exposed to single bacteria or to their cocktail (*p* < 0.05).

Furthermore, one-step growth curve analysis was performed to objectively compare the fecundity of KFS-EC3 phage with other reported broad- and narrow-spectrum phages infecting *E*. *coli* (Table S1). The lytic cycle of the phages was determined by measuring the adsorption time, eclipse time, latent time, and burst size. Because phage efficiency refers to the capability of lysing the target bacteria within a short period of time, KFS-EC3 exhibited relatively shorter eclipse (5 min) and latent times (20 min). However, its burst size (71 PFU/cell) was considerably smaller than that of other phages, including narrow CBA120 (440 PFU/cell) [59], narrow vB_EcoS_B2 (224 PFU/cell) [60], and polyvalent phiC119 (210 PFU/cell) [56] while it was similar to that of other polyvalent phages, including polyvalent SFP10 (100 PFU/cell) [11] and polyvalent PEf1 (99 PFU/cell) [55]. Although more studies are required to better understand phage dynamics, it can be suggested that the relatively shorter infection time that was observed will accelerate the lysis of target pathogens.

Since phages may be exposed to adverse environmental conditions during the processing, storage, and distribution of the food, their physiological stability is important. In particular, pH and temperature conditions will affect the lytic activity and stability of phages [44]. KFS-EC3 showed a greater stability in a wider pH range of 3–11 compared to that observed in polyvalent SFP10 phage [11] and polyvalent PS5 phage [44], having a pH range of 4–10. However, our phage was sensitive to extremely acidic (pH of 1 and 2) and alkaline (pH of 12) conditions. In addition, the greater stability of KFS-EC3 when exposed to a wide temperature range (between −70 °C and 60 °C) was similar to that observed in the other polyvalent phages SFP10 [11] and PS5 [44], which can withstand temperatures as high as 60 °C. Similar to polyvalent phage HY01 [9], KFS-EC3 lost the lytic activity at 60 °C. 

The efficiency of KFS-EC3 as a biocontrol agent was further evaluated using the in vitro bacterial challenge test. KFS-EC3 was shown to inhibit the growth of the indicator strain, *E. coli* O157:H7 ATCC 10536, effectively for 52 h and even showed the largest reduction of 2 log CFU/mL at the lowest MOI of 0.001. More importantly, the bactericidal effect was further sustained throughout the whole incubation period. Compared with other polyvalent phages reported, KFS-EC3 exhibited a relatively long duration time (52 h) when compared to HY01 (8 h) [9], SFP10 (10 h) [11], PEf1 (12 h) [55], and PS5 (24 h) [44]. However, other polyvalent phages exhibited a bacterial reduction of ~4 log CFU/mL with HY01 at MOI of 100 [9], SFP10 at MOI of 10 [11], and PS5 at MOI of >10,000 [44]). Although the bacterial reduction (~2 log CFU/mL) of KFS-EC3 was smaller than that of the above phages, our phage inhibited bacterial growth (~2 log CFU/mL) at the lowest MOI of 0.001 for a long duration. This is considered desirable as foodborne pathogens naturally exist in foods at very low concentrations [44]. A lower MOI has been reported to be more beneficial and economical for applications in foods [20]. Thus, KFS-EC3 may be an efficient biocontrol agent in various applications in foods that require a long period of processing and preservation.

Finally, the novelty and safety of KFS-EC3 was established through its genomic analysis. The novelty of KFS-EC3 was investigated based on whole-genome sequencing, phylogenetic analysis, and comparative analysis with similar phages. The complete genome of KFS-EC3 was compared with 20 phylogenetically related phages such as ime09 (98.04% identity), vB_EcoM_G50 (97.74% identity), vB_EcoM_IME339 (97.71% identity), Shfl2 (97.47% identity), and T4 phage (96.23% identity) (Table S2). Following the phylogenetic analysis with related phages and type phages based on assessment of the complete genome, capsid protein, and terminase large subunit, KFS-EC3 was classified as a member of the *Tequatrovirus* genus in the *Myoviridae* family. Furthermore, a comparative analysis with vB_EcoM_IME339 and T4 revealed KFS-EC3 as a newly isolated representative of the *Tequatrovirus* genus. The phage’s homologous region was associated with the tail fiber protein, which was considered to be the host receptor and could affect host specificity [61]. It was presumed to be the basis for a different polyvalent host spectrum of KFS-EC3. In addition, KFS-EC3 revealed the absence of genes associated with antibiotic resistance, virulence, allergenicity, and lysogenic factors already reported in relevant databases, thus suggesting its potential safety. In summary, the genetic analysis of KFS-EC3 revealed that this lytic phage is a newly isolated representative of the *Tequatrovirus* genus that is polyvalent and may be safe for its application as a biocontrol agent.

## 5. Conclusions

This study characterized a lytic phage, KFS-EC3, isolated from a slaughterhouse sewage in the aspect of effectiveness for using as a polyvalent and efficient phage. KFS-EC3 showed a polyvalent host spectrum capable of efficiently infecting *E. coli* O157:H7, *S. sonnei*, and *Salmonella* spp. In addition, it exhibited a high reproductive fecundity with rapid adsorption, short eclipse and latent period, and large burst size, as well as pH and temperature stability over a wide range. Genome analysis of KFS-EC3 revealed that it is a new phage of *Tequatrovirus,* and its potential safety was based on the absence of genes involved in virulence, allergenicity, lysogenicity, and antibiotic resistance. Notably, KFS-EC3 exhibited high efficiency by controlling *E*. *coli* O157:H7 for a long time with the lowest MOI. Thus, KFS-EC3 is a promising, cost-effective, and efficient biocontrol agent to be applied for the control of major foodborne pathogens in foods. Further studies should focus on investigating the phage receptors across various host bacteria which limits the wider infection range of KFS-EC3, including the bacterial defense system. In addition, it would be desirable to apply KFS-EC3 during the processing and preservation stages of foods which have various matrix for the simultaneous and efficient biocontrol of *E. coli* O157:H7, *S*. *sonnei*, and *Salmonella* spp.

## Figures and Tables

**Table 1:** Specificity and efficiency of plating of KFS-EC3.

Bacterial Strains	Clear Zone Formation ^a^	EOP ^b^	Source ^c^
*Aeromonas hydrophila* ATCC 7966	–	NT	ATCC
*A. hydrophila* JUNAH	–	NT	VMRI
*A. hydrophila* SNUFPC A3	–	NT	VMRI
*A. hydrophila* SNUFPC A5	–	NT	VMRI
*A. hydrophila* SNUFPC A7	–	NT	VMRI
*A. hydrophila* SNUFPC A9	–	NT	VMRI
*A. hydrophila* SNUFPC A10	–	NT	VMRI
*A. hydrophila* SNUFPC A11	–	NT	VMRI
*Bacillus cereus* ATCC 13061	–	NT	ATCC
*B. cereus* ATCC 14579	–	NT	ATCC
*B. cereus* ATCC 21768	–	NT	ATCC
*B. cereus* ATCC 1611	–	NT	ATCC
*B. subtilis* ATCC 6633	–	NT	ATCC
*Escherichia coli* O157:H7 ATCC 10536	+	1.00 ± 0.00	ATCC
*E. coli* O157:H7	+	0.91 ± 0.03	DPFS
*E. coli* O157:H7 204p	+	0.94 ± 0.01	DPFS
*E. coli* BW 25113	–	NT	DPFS
*E. coli* K12 ER2738	–	NT	DPFS
*E. coli* K12 VSM 1692	–	NT	DPFS
*E. coli* ATCC BAA-2196	–	NT	ATCC
*E. coli* ATCC 700599	–	NT	ATCC
*E. coli* ATCC 15144	–	NT	ATCC
*E. coli* ATCC BAA-2192	–	NT	ATCC
*Klebsiella pneumoniae* ATCC 13883	–	NT	ATCC
*Listeria monocytogenes* ATCC 7644	–	NT	ATCC
*L. monocytogenes* ATCC 19111	–	NT	ATCC
*L. monocytogenes* G3982 4b	–	NT	DPFS
*L. monocytogenes* G6055	–	NT	DPFS
*L. monocytogenes* H7738	–	NT	DPFS
*L. monocytogenes* H7757	–	NT	DPFS
*Pseudomonas aeruginosa* ATCC 10145	–	NT	ATCC
*Salmonella* Dublin	–	NT	DPFS
*S.* Enteritidis ATCC 13076	+	0.52 ± 0.02	ATCC
*S.* Hartford	–	NT	DPFS
*S.* Heidelberg	–	NT	DPFS
*S.* Mission	+	0.81 ± 0.01	DPFS
*S.* Montevideo	–	NT	DPFS
*S.* Newport	–	NT	DPFS
*S.* Salamae	–	NT	DPFS
*S.* Senftenberg	+	0.72 ± 0.05	DPFS
*S.* Typhi	–	NT	DPFS
*S.* Typhimurium ATCC 13311	–	NT	ATCC
*S.* Typhimurium NCTC 12023	–	NT	NCTC
*S.* Panama	–	NT	DPFS
*Shigella boydii* NCCP 11190	–	NT	NCCP
*S. flexneri* 2a 2457T	–	NT	DPFS
*S. sonnei* ATCC 9290	+	0.85 ± 0.02	ATCC
*Staphylococcus aureus* ATCC 25923	–	NT	ATCC
*S. aureus* p01115	–	NT	KNUHPRB
*S. aureus* p01328	–	NT	KNUHPRB
*S. aureus* p03020	–	NT	KNUHPRB
*S. aureus* p05182	–	NT	KNUHPRB
*Vibrio parahaemolyticus* ATCC 17802	–	NT	ATCC
*V. vulnificus*	–	NT	DPFS
*Yersinia enterocolitica* ATCC 23715	–	NT	ATCC
*Y. enterocolitica* ATCC 55075	–	NT	ATCC
*Y. enterocolitica* ATCC 9610	–	NT	ATCC

EOP: efficiency of plating. a +, clear zone formation; −, no clear zone formation b NT, Not Tested c ATCC, American Type Culture Collection; VMRI, College of Veterinary Medicine and Research Institute for Veterinary Science at Seoul National University; NCCP, National Culture Collection for Pathogens; NCTC, National Collection of Type Cultures; DPFS, Department of Plant and Food Sciences at Sangmyung University, Korea; KNUHPRB, Kyungpook National University Hospital Pathogen Resource Bank.

## Data Availability

Not applicable.

## Supplementary Materials

The following are available online at https://www.mdpi.com/article/10.3390/microorganisms9102105/s1, Figure S1: Plaque morphology of KFS-EC3 against (A) the indicator strain, (B) Shigella sonnei, and (C) Salmonella Mission. Figure S2: Adsorption assay of KFS-EC3. Figure S3: Polyvalent lytic capacity of KFS-EC3 after its exposure to each bacterium and their cocktail at an MOI of 1.0. Table S1: Several reported broad and narrow spectrum phages infecting *E. coli.* Table S2: General genomic characteristics of KFS-EC3 and phylogenetically related phages. Supplementary method S1: Adsorption assay of KFS-EC3. Supplementary method S2: Polyvalent lytic capacity of KFS-EC3 against various host strains.
