# Supplementary material for: Characterization of a New and Efficient Polyvalent Phage Infecting E. coli O157:H7, Salmonella spp., and Shigella sonnei
Source: Microorganisms. 2021 Oct 6;9(10):2105. doi: 10.3390/microorganisms9102105 (PMC8540833; doi:10.3390/microorganisms9102105)
Supplement: Supplementary file 1 [file microorganisms-09-02105-s001.zip › microorganisms-1396252-supplementary.pdf]

Supplementary method S1

Adsorption assay of KFS-EC3

Adsorption assay of KFS-EC3 was performed as previously described by Kim et al. (2018) with minor modification [1]. Briefly, KFS-EC3 was mixed with a culture of *E. coli* O157:H7 ATCC 10536 (10^8 CFU/mL) at an equivalent volume ratio to obtain a multiplicity of infection (MOI) of 0.001 and incubated at 37 °C for 8 min at 110 rpm. A 1 mL aliquot of the mixture was collected at every 2-min interval and centrifuged at 12,000 × *g* for 30 s at 4 °C. The supernatant containing the non-adsorbed phage was immediately filtered using a 0.20-μm cellulose acetate filter and the filtrate that was obtained was used for phage titer determination using a plaque assay as described in section 2.2. The experiment was conducted in triplicates and the adsorption rate was calculated using the following equation:
Adsorption rate (%) = (average phage titer at each time interval) / (average phage titer at time 0) × 100

Supplementary method S2

Polyvalent lytic capacity of KFS-EC3 against various host strains

For comparing the polyvalent lytic capacity of KFS-EC3 phage against each target bacterium and bacterial cocktail, KFS-EC3 (10^7 PFU/mL) was added to each bacterial suspension of *E. coli* O157:H7 ATCC 10536, *Salmonella* Mission, and *Shigella sonnei* ATTC 9290 and the cocktail (1:1:1, v/v) at an MOI of 1.0. SM buffer was added in the control instead of using the phage. Following incubation at 37 °C with gentle shaking for 4 h, the bacterial number was counted using xylose lysine deoxycholate (XLD, Difco Laboratories Inc.) and eosin methylene blue (EMB, Difco Laboratories Inc.) agar plates for comparison.

[Supplementary data]

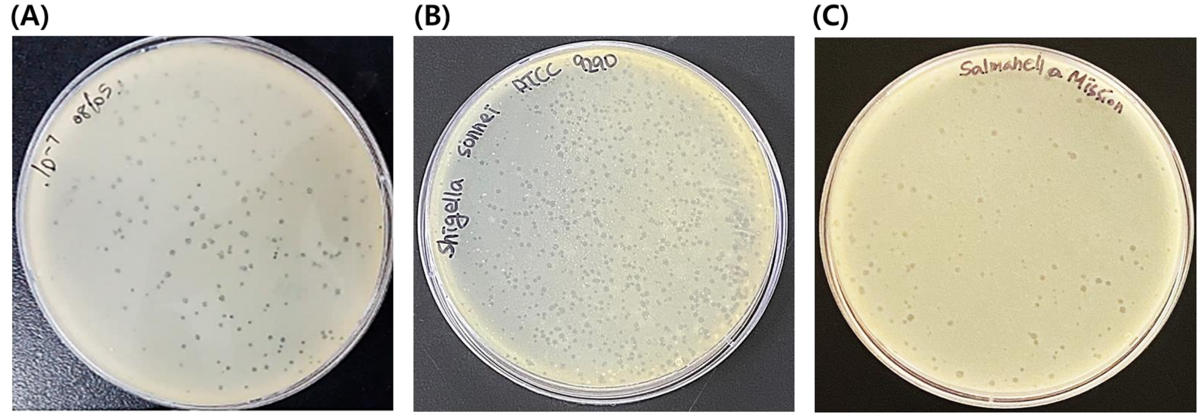

Figure S1. Plaque morphology of KFS-EC3 against (**A**) the indicator strain, (**B**) *Shigella sonnei*, and (**C**) *Salmonella* Mission.

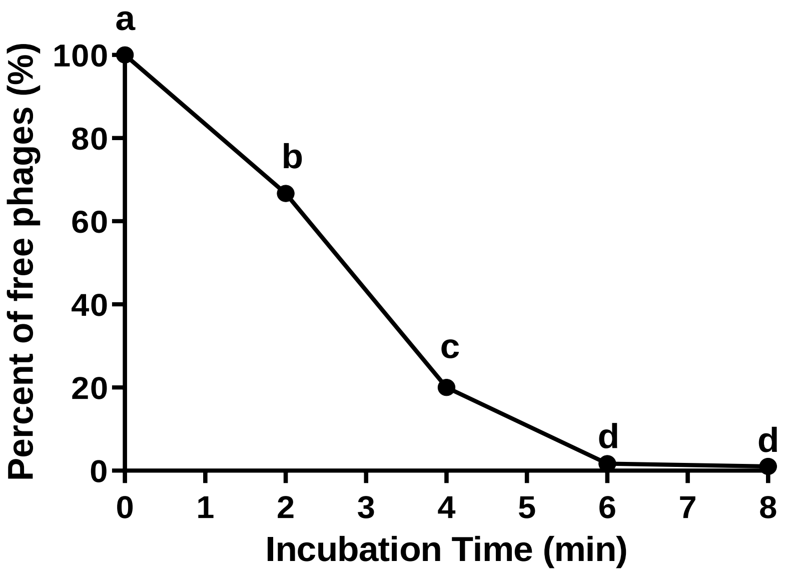

Figure S2. Adsorption assay of KFS-EC3.

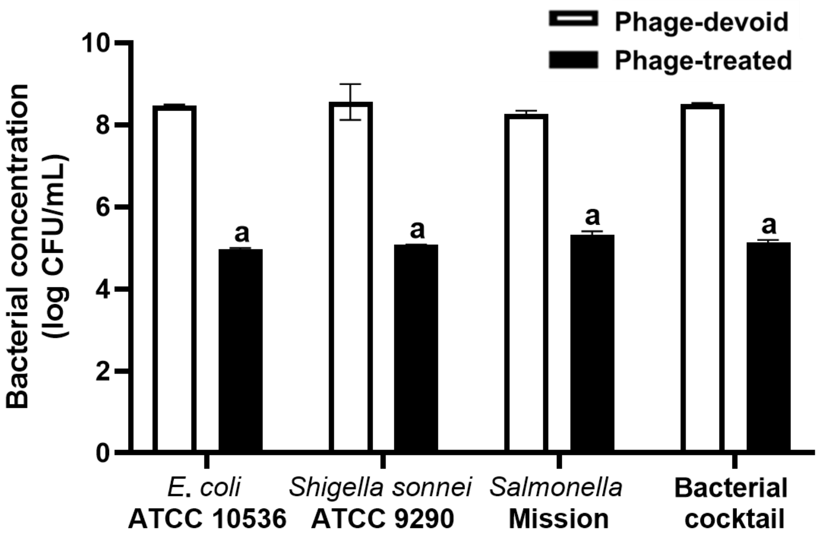

Figure S3. Polyvalent lytic capacity of KFS-EC3 after its exposure to each bacterium and their cocktail at an MOI of 1.0.

Table S1. Several reported broad and narrow spectrum phages infecting *E. coli*

Phage	Host strain	Classification	Size of length (nm)		Specificity	Eclipse period (min)	Latent period (min)	Burst size (PFU/cell)	Reference
			Head	Tail					
**KFS-EC3**	***E. coli* O157:H7 ATCC 10536**	***Myoviridae***	**96.35**	**68.42–133.48**	**Polyvalent (3 genera)**	**5**	**20**	**71**	**This study**
SFP10	*E. coli* O157:H7 ATCC 43890	*Myoviridae*	68.75	41.67–131.25	Polyvalent (2 genera)	15	25	100	[2]
HY01	*E. coli* O157:H7 ATCC 43890	*Myoviridae*	100	100	Polyvalent (2 genera)	5	25	25	[3]
PS5	*S.* Typhimurium NBRC12529	*Myoviridae*	84	106	Polyvalent (2 genera)	-	25	204	[4]
PEf1	*E. coli* K-12	*Siphoviridae*	-	-	Polyvalent (2 genera)	-	50	99	[5]
phiC119	*E. coli* O157 EC-48	*Siphoviridae*	43	168	Polyvalent (2 genera)	-	20	210	[6]
SH6	*Shigella flexneri* SF1	*Siphoviridae*	62 ± 2	161 ± 2	Polyvalent (2 genera)	-	16	103 ± 16	[7]
vB_EcoM_swi3	*E. coli* K88	*Myoviridae*	80	120	Polyvalent (2 genera)	-	25	25	[8]
EcS1	*E. coli* BL21	*Myoviridae*	111 ± 5.5	110 ± 5.5	Polyvalent (3 genera)	-	-	-	[9]
SH7	*S. flexneri* SF1	*Myoviridae*	112 ± 5	116 ± 4	Polyvalent (3 genera)	-	23	26 ± 5	[7]
T4 *Yer* mutants	*E. coli* K-12	*Myoviridae*	-	-	Polyvalent (3 genera)	-	-	-	[10]
KFS-EC	*E. coli* O157:H7 ATCC 43895	*Myoviridae*	100	113	Narrow	5	30	150	[11]
CBA120	*E. coli* O157:H7 NCTC 12900	*Myoviridae*	90	105	Narrow	20	40	440	[12]
CEV1	*E. coli* O157:H7 NCTC 12900	*Myoviridae*	100	100	Narrow	18	26	150	[13]
FAHEc1	*E. coli* O157:H7 isolate ERL 022447	*Myoviridae*	99	108	Narrow	-	-	-	[14]
rV5	*E. coli* O157:H7 strain R508	*Myoviridae*	91	121	Narrow	-	-	-	[15]
vB_EcoS_ACG-C40	*E. coli* isolate Can 40	*Myoviridae*	110	114	Narrow	-	-	-	[16]
vB_EcoS_ACG-C91	*E. coli* isolate Can 91	*Podoviridae*	65–68	12	Narrow	-	-	-	[16]
vB_EcoS_ACG-M12	*E. coli* isolate MSHS1210	*Siphoviridae*	57	172	Narrow	-	-	-	[16]
Φ241	*E. coli* O157:H7 strain B0241	*Myoviridae*	80	33	Narrow	-	15	53	[17]
vB-EcoS_Rogue1	*E. coli* O157:H7 R508N	*Siphoviridae*	53	152	Narrow	-	-	-	[18]
vB_EcoS_HSE2	*E. coli* strain 40371	*Siphoviridae*	56	178	Narrow	-	30	86	[19]
Vb_EcoS_B2	*E. coli* BL21	*Siphoviridae*	48	143	Narrow	-	30	224	[20]
vB_EcoM_IME339	*E. coli* BL21	*Myoviridae*	-	-	-	-	-	91	[21]
Teqskov	*E. coli* K-12 MG1655	*Myoviridae*	-	-	-	-	-	-	[22]
Teqhal	*E. coli* K-12 MG1655	*Myoviridae*	-	-	-	-	-	-	[22]
Slur08	*E. coli* K-12 MG1655	*Myoviridae*	-	-	-	-	-	-	[23]
Slur03	*E. coli* K-12 MG1655	*Myoviridae*	-	-	-	-	-	-	[23]
phiLLS	*E. coli* O157:H7 CECT 4076	*Siphoviridae*	56	135	-	-	70	176	[24]

Table S2. General genomic characteristics of KFS-EC3 and phylogenetically related phages

Phage	Host	Genome (bp)	GC content (%)	Taxonomy (Family; Genus)	Identity (%)	Accession number
KFS-EC3	*E. coli* O157:H7 ATCC 10536	166,440	35.5	*Myoviridae; Tequatrovirus*	-	MZ065353
Slur13	*E. coli* K-12 MG1655	167,299	35.5	*Myoviridae; Tequatrovirus*	96.24	LN881737.1
Slur04	*E. coli* K-12 MG1655	167,298	35.5	*Myoviridae; Tequatrovirus*	96.24	NC042130.1
Slur11	*E. coli* K-12 MG1655	167,298	35.5	*Myoviridae; Tequatrovirus*	96.24	LN881734.1
Teqhal	*E. coli* MG1655 K-12	168,070	35.1	*Myoviridae; Tequatrovirus*	96.28	MN895435.1
Teqsoen	*E. coli* K-12 MG1655	166,468	35.5	*Myoviridae; Tequatrovirus*	96.00	MN895436.1
Kha5h	*E. coli* O103:H2	167,318	35.5	*Myoviridae; Tequatrovirus*	96.35	KT184312.1
Shfl2	*Shigella flexneri*	165,919	35.6	*Myoviridae; Tequatrovirus*	97.47	HM035025.1
ime09	*E. coli*	166,499	35.7	*Myoviridae; Tequatrovirus*	98.04	JN202312.1
vB_EcoM_IME339	*E. coli* BL21	164,366	35.6	*Myoviridae; Tequatrovirus*	97.71	MH051915.1
vB_EcoM_G50	*E. coli*	167,728	35.5	*Myoviridae; Tequatrovirus*	97.74	MK327942.1
vB_EcoM_KAW1E185	*E. coli*	164,987	35.4	*Myoviridae; Tequatrovirus*	96.56	MK373781.1
Teqskov	*E. coli* MG1655 K-12	165,017	35.4	*Myoviridae; Tequatrovirus*	96.69	MN895437.1
phiD1	*Yersinia pestis*	167,063	35.5	*Myoviridae; Tequatrovirus*	96.81	HE956711.1
vB_EcoM_Ozark	*E. coli* (STEC)	167,600	39.5	*Myoviridae; Tequatrovirus*	96.49	MT682713.1
Slur08	*E. coli* K-12 MG1655	167,467	35.4	*Myoviridae; Tequatrovirus*	96.12	LN881733.1
Slur14	*E. coli* K-12 MG1655	167,467	35.4	*Myoviridae; Tequatrovirus*	96.13	LN881736.1
Slur03	*E. coli* K-12 MG1655	167,467	35.4	*Myoviridae; Tequatrovirus*	96.12	NC042129.1
PhiZZ30	*Serratia* sp. ATCC 39006	167,484	35.3	*Myoviridae; Tequatrovirus*	96.25	MT176426.1
vB_EcoM_Lutter	*E. coli* (STEC)	170,054	35.4	*Myoviridae; Tequatrovirus*	96.39	MT682714.1
T4	*E. coli* B strain	168,908	35.3	*Myoviridae; Tequatrovirus*	96.23	MT984581.1

References

1. Kim, S.; Kim, S. H.; Rahman, M.; Kim, J. Characterization of a *Salmonella* Enteritidis bacteriophage showing broad lytic activity against Gram-negative enteric bacteria. *J. Microbiol.* **2018**, *56*, 917–925.
2. Park, M.; Lee, J. H.; Shin, H.; Kim, M.; Choi, J.; Kang, D. H.; Ryu, S. Characterization and comparative genomic analysis of a novel bacteriophage, SFP10, simultaneously inhibiting both *Salmonella* enterica and *Escherichia coli* O157: H7. *Appl. Environ. Microbiol.* **2012**, *78*, 58–69.
3. Lee, H.; Ku, H. J.; Lee, D. H.; Kim, Y. T.; Shin, H.; Ryu, S.; Lee, J. H. Characterization and genomic study of the novel bacteriophage HY01 infecting both *Escherichia coli* O157: H7 and *Shigella flexneri*: potential as a biocontrol agent in food. *PLoS One* **2016**, *11*, e0168985.
4. Duc, H. M.; Son, H. M.; Yi, H. P. S.; Sato, J.; Ngan, P. H.; Masuda, Y.; Miyamoto, T. Isolation, characterization and application of a polyvalent phage capable of controlling *Salmonella* and *Escherichia coli* O157: H7 in different food matrices. *Food Res. Int.* **2020**, *131*, 108977.
5. Yu, P.; Mathieu, J.; Li, M.; Dai, Z.; Alvarez, P. J. Isolation of polyvalent bacteriophages by sequential multiple-host approaches. *Appl. Environ. Microbiol.* **2016**, *82*, 808–815.
6. Amarillas, L.; Chaidez, C.; González-Robles, A.; Lugo-Melchor, Y.; León-Félix, J. Characterization of novel bacteriophage phiC119 capable of lysing multidrug-resistant Shiga toxin-producing *Escherichia coli* O157: H7. *PeerJ*, **2016**, *4*, e2423.
7. Hamdi, S.; Rousseau, G. M.; Labrie, S. J.; Tremblay, D. M.; Kourda, R. S.; Slama, K. B.; Moineau, S. Characterization of two polyvalent phages infecting *Enterobacteriaceae*. *Sci. Rep.* **2017**, *7*, 1–12.
8. Sui, B.; Han, L.; Ren, H.; Liu, W.; Zhang, C. A Novel Polyvalent Bacteriophage vB_EcoM_swi3 Infects Pathogenic *Escherichia coli* and *Salmonella enteritidis*. *Front. Microbiol.* **2021**, 1496.
9. Saad, A. M.; Askora, A.; Kawasaki, T.; Fujie, M.; Yamada, T. Full genome sequence of a polyvalent bacteriophage infecting strains of *Shigella*, *Salmonella*, and *Escherichia*. *Arch. Virol*. **2018**, *163*, 3207-3210.
10. Tétart, F.; Repoila, F.; Monod, C.; Krisch, H. M. Bacteriophage T4 host range is expanded by duplications of a small domain of the tail fiber adhesin. *J. Mol. Biol*. **1996**, 258, 726–731.
11. Lee, C.; Choi, I. Y.; Park, D. H.; Park, M. K. Isolation and characterization of a novel *Escherichia coli* O157: H7-specific phage as a biocontrol agent. *J. Environ. Health Sci. Eng.* **2020**, 1–11.
12. Kutter, E. M.; Skutt-Kakaria, K.; Blasdel, B.; El-Shibiny, A.; Castano, A.; Bryan, D.; Brabban, A. D. Characterization of a ViI-like phage specific to *Escherichia coli* O157: H7. *Virol. J.* **2020**, *8*, 1–14.
13. Raya, R. R.; Varey, P.; Oot, R. A.; Dyen, M. R.; Callaway, T. R.; Edrington, T. S.; Kutter E. M.; Brabban, A. D. Isolation and characterization of a new T-even bacteriophage, CEV1, and determination of its potential to reduce *Escherichia coli* O157: H7 levels in sheep. *Appl. Environ. Microbiol*. **2006**, *72*, 6405–6410.
14. Hudson, J. A.; Billington, C.; Cornelius, A. J.; Wilson, T.; On, S. L. W.; Premaratne, A.; King, N. J. Use of a bacteriophage to inactivate *Escherichia coli* O157: H7 on beef. *Food Microbiol*. **2013**, *36*, 14–21.
15. Kropinski, A. M.; Waddell, T.; Meng, J.; Franklin, K.; Ackermann, H. W.; Ahmed, R.; Mazzocco, A.; Yates, J.; Lingohr E. J.; Johnson, R. P. The host-range, genomics and proteomics of *Escherichia coli* O157: H7 bacteriophage rV5. *Virol. J.* **2013**, *10*, 1–12.
16. Chibeu, A.; Lingohr, E. J.; Masson, L.; Manges, A.; Harel, J.; Ackermann, H. W.; Kropinski, A. M.; Boerlin, P. Bacteriophages with the ability to degrade uropathogenic *Escherichia coli* biofilms. *Viruses* **2012**, *4*, 471–487.
17. Lu, Z.; Breidt, F. *Escherichia coli* O157: H7 bacteriophage Φ241 isolated from an industrial cucumber fermentation at high acidity and salinity. *Front. Microbiol*. **2015**, *6*, 67.
18. Kropinski, A. M.; Lingohr, E. J.; Moyles, D. M.; Ojha, S.; Mazzocco, A.; She, Y. M.; Bach, S. J.; Rozema, E. A.; Stanford, K.; McAllister, T. A.; Johnson, R. P. Endemic bacteriophages: a cautionary tale for evaluation of bacteriophage therapy and other interventions for infection control in animals. *Virol. J.* **2012**, *9*, 1-9.
19. Peng, Q.; Yuan, Y. Characterization of a newly isolated phage infecting pathogenic *Escherichia coli* and analysis of its mosaic structural genes. *Sci. Rep*. **2018**, *8*, 1–10.
20. Xu, Y.; Yu, X.; Gu, Y.; Huang, X.; Liu, G.; Liu, X. Characterization and genomic study of phage vB_EcoS-B2 infecting multidrug-resistant *Escherichia coli*. *Front. Microbiol*. **2018**, *9*, 793.
21. Li, P.; Lin, H.; Mi, Z.; Xing, S.; Tong, Y.; Wang, J. Screening of polyvalent phage-resistant *Escherichia coli* strains based on phage receptor analysis. *Front. Microbiol*. **2019**, *10*, 850.
22. Olsen, N. S.; Forero-Junco, L.; Kot, W.; Hansen, L. H. Exploring the remarkable diversity of culturable *Escherichia coli* phages in the Danish Wastewater Environment. *Viruses* **2020**, *12*, 986.
23. Smith, R.; O'Hara, M.; Hobman, J. L.; Millard, A. D. Draft genome sequences of 14 *Escherichia coli* phages isolated from cattle slurry. *Genome Announc*. **2015**, *3*, e01364–15.
24. Amarillas, L.; Rubí-Rangel, L.; Chaidez, C.; González-Robles, A.; Lightbourn-Rojas, L.; León-Félix, J. Isolation and characterization of phiLLS, a novel phage with potential biocontrol agent against multidrug-resistant *Escherichia coli*. *Front. Microbiol*. **2017**, 8, 1355.
